# Volatile Metabolomic Composition of *Vitex* Species: Chemodiversity Insights and Acaricidal Activity

**DOI:** 10.3389/fpls.2017.01931

**Published:** 2017-11-14

**Authors:** José G. de Sena Filho, Ighor C. Barreto, Avaldo O. Soares Filho, Paulo C. L. Nogueira, Adenir V. Teodoro, Ana V. Cruz da Silva, Haroudo S. Xavier, Allívia R. C. Rabbani, Daniel J. Spakowicz, Jennifer M. Duringer

**Affiliations:** ^1^Empresa Brasileira de Pesquisa Agropecuária – EMBRAPA Tabuleiros Costeiros, Aracaju, Brazil; ^2^Herbário HUESBVC, Departamento de Ciências Naturais, Universidade Estadual do Sudoeste da Bahia, Conquista, Brazil; ^3^Laboratório de Pesquisa em Química Orgânica de Sergipe, Departamento de Química – CCET, Universidade Federal de Sergipe, São Cristóvão, Brazil; ^4^Departamento de Ciências Farmacêuticas, Universidade Federal de Pernambuco, Recife, Brazil; ^5^Bahia Federal Institute of Education, Science and Technology – IFBA, Porto Seguro, Brazil; ^6^Program in Comparative Biology and Bioinformatics, Yale University, New Haven, CT, United States; ^7^Department of Environmental and Molecular Toxicology, Oregon State University, Corvallis, OR, United States

**Keywords:** *Vitex capitata*, Vitex megapotamica, Vitex gardneriana, Vitex rufescens, chemodiversity

## Abstract

The *Vitex* genus (Lamiaceae) produces a plethora of metabolites that include ecdysteroids and terpenoids, some of which have demonstrated insect repellent properties. The volatile composition of several members of this genus has not been chemically defined, as many taxa are endemic to remote ecosystems. In this study, leaves were collected from the northeast of Brazil from *Vitex capitata, V. megapotamica, V. gardneriana*, and *V. rufescens* plants and examined for their chemical profile via GC-MS/FID of essential oil extracts. The analyses showed a diversity of terpenoids. Of particular note were seven-member ring sesquiterpenes which were present in great abundance; a dendrogram showed clades separating by the production of bicyclogermacrene, aromadendrane and 5,10-cycloaromadendrane sesquiterpenoids for the four species. Comparison of volatile metabolite profiles to 13 other *Vitex* species showed strong similarities in the production of some monoterpenes, but varied by their production of larger terpenes, especially those with gem-dimethylcyclopropyl subunits on seven-member ring compounds. From this work, we suggest that the sesquiterpene skeleton with seven member rings is a good chemosystematic biomarker candidate for the *Vitex* genus. Separation using this biomarker was then validated using Inter-Simple Sequence Repeat profiling. Lastly, experiments examining the toxicity of these four oils against the coconut mite *Aceria guerreronis* showed that only the oil of *V. gardneriana* had strong acaricidal activity, with an LC_50_ of 0.85 mg/mL, thus demonstrating its potential for use as a natural pesticide.

## Introduction

Metabolomic profiles from organisms in the Plantae and other kingdoms have been used to distinguish samples from different species and ecotypes, as well as aid in their classification (Dettmer et al., 2007; Allwood et al., 2011; Aliferis et al., 2013; Jing et al., 2015). In the case of plants, much attention has been given to one of the largest group of metabolites they produce, the terpenoids. In a study similar to ours which explored the chemodiversity of *Selaginella* in the context of evolution, Weng and Noel (2013) suggest that monoterpene and sesquiterpene synthases are derived from conserved diterpene synthases, based off of phylogenetic analyses of plant terpene synthases done by Bohlmann et al. (1998). Thus, it appears that monoterpenes and sesquiterpenes are frequently used as chemical markers for distinguishing between genera. For example, in the *Lantana* (Verbenaceae) genus, β-caryophyllene was the major compound suggested as a chemical marker (Sena Filho et al., 2010), while in *Citrus*, both cyclic (*R*-(+)-limonene and α-terpinene) and bicyclic monoterpenes (*α*-pinene and sabinene) were dominant in fruit essential oils and therefore proposed as markers for those species/varieties (Jing et al., 2015). Worldwide, the volatile metabolomics composition of *Vitex* species has been poorly investigated, likely due to their accessibility and phenotype, namely, their size and bad odor. From a total of 250 species, only about 35 are reported in the literature with in-depth chemical characterizations. Many of them are difficult to identify due to endemism, e.g., *Vitex rufences* A. Juss, *Vitex capitata* Vahl, *Vitex gardneriana* Schauer, which are found only in areas in the northeast of Brazil.

The main goal of this study was to develop a metabolomics approach for evaluating the volatile composition of *Vitex* species, using four species we collected (*Vitex capitata* Vahl, *Vitex megapotamica* (Spreng.) Moldenke, *Vitex gardneriana* Schauer and *Vitex rufescens* A. Juss) in addition to 13 *Vitex* species published in the literature. First, the chemical composition of the volatile fraction of essential oils from leaves of these species was characterized via GC-MS/FID. Subsequently, cluster analyses of the chemical composition of the 17 *Vitex* species were performed. Next, Inter-Simple Sequence Repeat (ISSR) was used to evaluate the genetic variation between our four *Vitex* species, followed by cluster analyses. Lastly, bioactivity of our four essential oil extracts was evaluated using toxicity assays against *Aceria guerreronis* Keifer (Acari: Eriophyidae), a major pest found in coconut plantations worldwide (Návia et al., 2013). From this study, we aimed to chemosystematically characterize the *Vitex* genus, in addition to expand our understanding of it in an evolutionary context.

## Materials and Methods

### Plant Material

Leaves from *V. capitata, V. megapotamica, V. gardneriana*, and *V. rufescens* were collected in the northeast of Brazil during the rainy season in June of 2015. Determination of species was carried out by Prof. Dr. Avaldo de Oliveira Soares Filho, by comparison to exsiccates from the Universidade Estadual do Sudoeste da Bahia (HUESBVC) and specimens of virtual herbaria, in addition to use of technical bibliographies (Salimena-Pires and Giulietti, 1998; Barroso et al., 1999; Lima and Rança, 2009). Genus of taxonomic classification followed the Angiosperm Phylogenetic Group (APG III) guidelines. Vouchers of the specimens were deposited in the Herbarium of HUESVC in Vitoria da Conquista, Brazil, under the numbers 8194 (*V. capitata*), 8195 (*V. megapotamica*) and 8126 (*V. gardneriana*). *V. rufescens* was deposited under number 38352 in the Herbarium of the Universidade Federal de Sergipe (UFS) in Aracaju.

### Essential Oil Isolation Procedure

Essential oils from fresh leaves (1 kg) were extracted via hydrodistillation for 4 h with a Clevenger-type apparatus (Sena Filho et al., 2010). The extracted oils were dried with powdered anhydrous sodium sulfate and kept at 4°C in a sealed amber bottle before analysis by GC-MS/FID.

### GC-MS/FID Analysis

Essential oil characterization was performed using methods described previously (Bittrich et al., 2013; Ramos et al., 2013; Feitosa-Alcantara et al., 2017). Specifically, a GC-MS/FID (QP2010 Ultra, Shimadzu Corporation, Kyoto, Japan) equipped with an autosampler (AOC-20i, Shimadzu) was used to separate compounds with an Rtx^®^-5MS Restek fused silica capillary column (5%-diphenyl–95%-dimethyl polysiloxane) of 30 m × 0.25 mm i.d., 0.25 μm film thickness, at a constant helium (99.999% purity) flow rate of 1.2 mL⋅min^-1^. An injection volume of 0.5 μL was employed, with a split ratio of 1:10. The oven temperature program started at 60°C, was held for 3 min, then increased at a rate of 5°C/min to 300°C, and was finally held for 9 min. The MS and FID data were simultaneously acquired by employing a detector splitting system, with a split flow ratio of 5:1 (MS:FID). A 0.4 m × 0.15 mm i.d. restrictor tube (capillary column) was used to connect the splitter to the MS detector, while a 0.6 m × 0.22 mm i.d. restrictor tube was used to connect the splitter to the FID detector. The MS data were acquired in full scan mode (*m/z* of 40-550) at a scan rate of 0.3 scan/s using an electron ionization potential of 70 eV. The injector temperature was set to 280°C and the ion source temperature to 200°C. The FID temperature was 300°C, and the gas supplies for the FID were hydrogen, air, and helium at flow rates of 30, 300, and 30 mL⋅min^-1^, respectively. Quantification of each constituent was estimated by FID peak-area normalization (%). Compound concentrations were calculated from the GC peak areas relative to the total peak area of all peaks and were arranged in order of GC elution. The retention index was obtained by co-injecting the oil sample with a C7–C30 linear hydrocarbon mixture; identification was made based on comparison of retention indices and fragmentation patterns with published values (Linstrom and Mallard, 2005; Adams, 2007).

### Cluster Analyses of Essential Oil Volatile Metabolomic Composition

Molecules comprising at least 2% of the total FID signal were summarized for the four *Vitex* species from this study as well as 13 species referenced in the literature (**Supplementary Table S1**). The cluster analysis was then carried out using the Euclidean Distance and the Unweighted Pair Group Method with Arithmetic Mean (UPGMA) cluster algorithm (Sokal and Michener, 1958). Statistical analysis was performed using the Paleontological Statistics Software Package for Education and Data Analysis (PAST) (Hammer et al., 2001). Dendrograms were then generated which assigned diversity of the 17 *Vitex* species based on their essential oil components.

### Genetic Analysis with ISSR Markers

The four *Vitex* species we collected (*Vitex capitata, V. megapotamica, V. gardneriana* and *V. rufescens*) were analyzed by repeat sequence primers via Inter-Simple Sequence Repeat (ISSR). The DNA was extracted from young, fresh leaves as previously described (Doyle, 1991). Fourteen primers were used to screen for polymorphisms (**Table 1**). The PCR amplification parameters and ISSR method used followed that as described in Sena Filho et al. (2010).

**Table 1:** Primer information for DNA markers used to screen for polymorphisms in four *Vitex* specimens gathered from northeastern Brazil.

Code^∗^	Forward primer (5′-3′)	Annealing Temp (°C)	NF	PIC	MI
809	AGAGAGAAGAGAGA GG	57.2°C	5	0.34	1.70
818	CACACACACACACACAG	57.2°C	6	0.41	2.46
823	TCTCTCTCTCTCTCTCC	57.2°C	8	0.23	1.86
826	ACACACACACACACACC	57.2°C	9	0.34	3.09
842	GAGAGAGAGAGAGAGAYG	58.8°C	4	0.44	3.05
843	CTCTCT CTCTCTCTCTRA	56.5°C	6	0.29	1.76
845	CTCTCTCTCTCTCTCTRG	58.8°C	2	0.23	0.46
848	CACACACACACACACCRG	58.8°C	6	0.35	2.11
855	ACACACACACACACACYT	56.5°C	7	0.28	1.99
856	ACACACACACACACACYA	56.5°C	9	0.35	3.17
857	ACACACACACACACYG	58.8°C	11	0.31	3.46
858	TGTGTGTGTGTGTGTGRT	56.5°C	7	0.30	2.07
888	BDBCACACACACACACA	56.5°C	2	0.32	0.65
890	VHVGTGTGTGTGTGTGT	56.5°C	8	0.18	1.43

*^∗^Codes represent primer number from the database at the University of British Columbia. NF, number of fragments; PIC, polymorphic information content and MI, Marker Index.*

### Cluster Analysis of Genetic Markers

Based on the presence or absence of fragments, the polymorphic information content (PIC) (Ghislain et al., 1999), the marker index (MI) (Zhao et al., 2007), the similarity coefficient matrix (Jaccard, 1908), and the clustering of the matrix were calculated for each primer using the UPGMA cluster algorithm (Sokal and Michener, 1958). The bootstrap method was used with 100,000 replicates by employing FreeTree software (Pavlicek et al., 1999) to generate the dendrogram (Page, 1996).

### Toxicity to *A. guerreronis*

Concentration-mortality bioassays (Silva et al., 2013) were conducted to estimate the lethal concentration (LC) of the essential oil extracted from four *Vitex* species (*V. capitata, V. megapotamica, V. gardneriana*, and *V. rufescens*) to adult coconut mites (*A. guerreronis*). A preliminary bioassay was performed using a wide concentration range where the no observed adverse effect level (NOAEL) and the concentration able to kill 100% of *A. guerreronis* were determined. Afterward, four concentrations within this range were used to generate a concentration response curve as follows: oil was sprayed onto perianth disks (1 cm diameter) of young coconut fruits placed in Petri dishes containing agar at 5%. Spraying of the oils was performed at a pressure of 34 kPa (0.34 bar) with a 9.3-ml spray rate using a Potter Tower device (Burkard, Rickmansworth, United Kingdom) (Oliveira et al., 2017). Sprayed perianth disks were allowed to dry for 20 min before 20 adult *A. guerreronis* were placed onto each disk. Control disks were sprayed with acetone, which was also used to dissolve and dilute the oils for all concentrations. Petri dishes were individually maintained in an incubator at 26°C and 70% relative humidity for 24 h. Eight replicates (wells) for each oil concentration were used. Harmfulness of the commercial acaricide abamectin (Vertimec 18 EC^TM^; 18 g a.i./L, at field dosage 75 mL/100 L) to *A. guerreronis* was evaluated as a way of toxicity comparison (20 replicates were performed and control disks were sprayed with distilled water). Mite mortality was assessed after 24 h of exposure and concentration-mortality curves were estimated by Probit analysis using the PROC PROBIT procedure in SAS (version 9.4, Cary, NC, United States). The likelihood ratio chi-square goodness-of-fit was applied to evaluate whether the data adequately conformed to the PROBIT model (Robertson et al., 2007).

## Results

**FIGURE 1:**
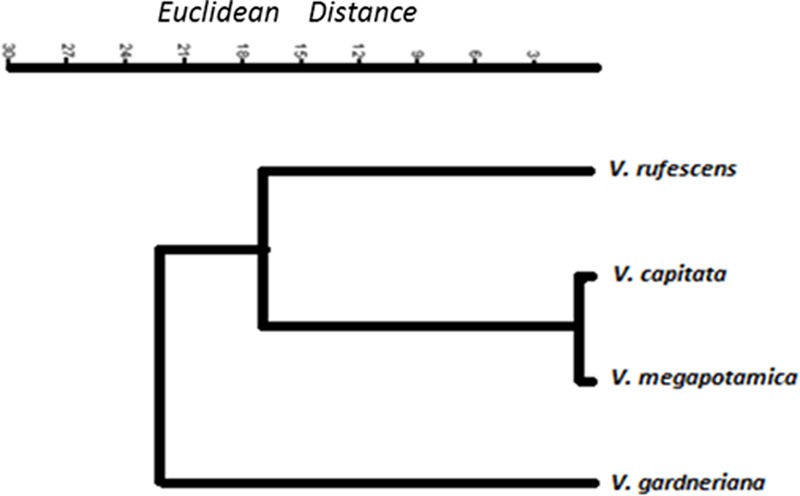
Dendrogram of sesquiterpenes containing seven-member rings found in the essential oil extract of four *Vitex* specimens collected in northeastern Brazil.

**FIGURE 2:**
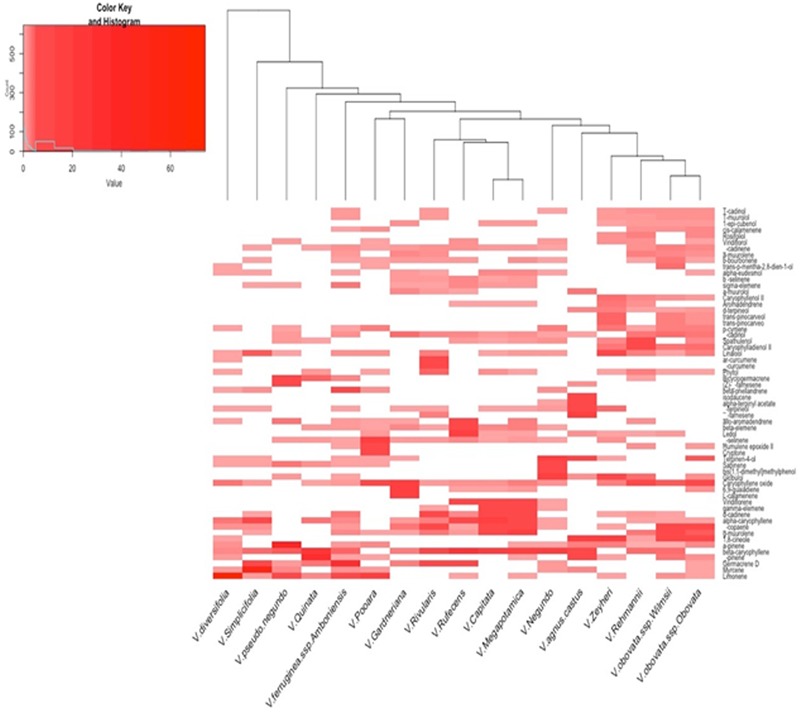
Heat map of the volatile compounds characterized from essential oil extracts via GC-MS FID of 17 *Vitex* species. Heat map was generated using Euclidean Distance and the Unweighted Pair Group Method with Arithmetic Mean cluster algorithm. Compounds representing at least 2% of the GC-FID signal were included (for a listing of compounds, see **Supplementary Table S1**).

GC-MS/FID analysis of essential oils collected from leaves of *V. capitata, V. megapotamica, V. gardneriana* and *V. rufescens* (yield of 0.13, 0.18, 0.33, and 0.45%, respectively) determined 46, 45, 41, and 37 constituents to be present, respectively (**Table 2**). Grouping for the species by Euclidian distance based on seven-member ring sesquiterpenes showed clades separating by the production of bicyclogermacrene, aromadendrane and 5,1-cycloaromadendrane sesquiterpenoids, e.g., aromadendrene, viridiflorene, ledol, viridiflorol, *allo*-aromadendrene, bicyclogermacrene, globulol, 6,9-guaiadiene, spathulenol and palustrol (**Figure 1**). We then analyzed the volatile composition of the essential oils from 13 previously published *Vitex* species, as well as the four *Vitex* species studied here, three of them endemic to Brazil (*V. capitata, V. gardneriana* and *V. rufescens*) via a clustering algorithm (**Figure 2**). The 17 species comparison showed strong similarities in the production of some monoterpenes, but varied by their production of larger terpenes, especially those with gem-dimethylcyclopropyl subunits on seven-member ring compounds.

**Table 2:** Major components (%) identified in the essential oil extracted from leaves of *Vitex capitata, V. megapotamica, V. gardneriana* and *V. rufescens* from northeastern Brazil^a^.

RI lit.^b^	RI exp.^c^	Compounds	*V. capitata*	*V. megapotamica*	*V. gardneriana*	*V. rufescens*
846	865	(2*E*)-Hexenal	–	–	–	tr^d^
850	866	(3*Z*)-Hexen-1-ol	–	–	–	0.4
863	876	Hexan-1-ol	–	–	–	tr
932	934	α-Pinene	–	0.3	–	tr
974	965	β-Pinene	–	0.4	–	–
974	981	Oct-1-en-3-ol	–	–	–	tr
1024	1020	Limonene	–	0.1	–	0.1
1067	1066	*cis*-Linalool oxide (furanoid)	–	0.1	–	–
1084	1083	*trans*-Linalool oxide (furanoid)	–	0.1	–	–
1095	1094	Linalool	0.2	1.1	tr	tr
1122	1132	α-Campholenal	–	–	tr	–
1135	1146	Nopinone	–	–	tr	–
1160	1171	Pinocarvone	–	–	0.1	–
1174	1185	Terpinen-4-ol	–	–	0.2	–
1184	1182	Neoisomentol	tr	–	–	–
1186	1196	α-Terpineol	0.1	0.2	0.1	–
1195	1205	Myrtenal	–	–	0.2	–
1284	1292	Dihydroedulan IIA	–	–	–	tr
1292	1297	Dihydroedulan IA	tr	–	–	tr
1298	1302	Theaspirane	–	–	–	tr
1315	1319	Theaspirane B	–	–	–	tr
1335	1341	δ-Elemene	0.7	1.0	0.5	1.8
1345	1353	α-Cubebene	1.8	1.9	1.0	tr
1369	1380	Cyclosativene	tr	–	–	–
1373	1376	α-Ylangene	0.1	0.1	–	tr
1374	1380	α**-Copaene**	11.7	10.8	3.2	0.3
1379	1384	β-Patchoulene	–	–	–	tr
1383	1393	(*E*)-β-Damascenone	tr	0.3	0.3	–
1387	1389	β-Bourbonene	tr	1.3	0.6	0.6
1389	1398	β-Elemene	2.2	2.7	1.9	5.8
1409	1416	α**-Gurjunene**	0.1	0.1	–	1.6
1417	1430	**(*E*)-Caryophyllene**	19.7	16.2	2.7	21.0
1430	1435	β-Copaene	–	–	0.5	0.8
1434	1445	γ**-Elemene**	6.7	5.6	–	–
1437	1451	α-Guaiene	0.2	0.3	0.1	–
1439	1456	Aromadendrene	0.3	0.2	–	0.3
1442	1460	(*Z*)-α-farnesene	^∗^	^∗^	–	–
1442	1461	**6,9-Guaiadiene**	0.3^∗^	0.8^∗^	19.3	0.3
1451	1466	*trans*-Muurola-3,5-diene	tr	0.3	1.8	–
1452	1473	α**-Humulene**	15.7	8.5	0.9	7.3
1458	1479	***allo*-Aromadendrene**	1.9	2.3	0.5	6.9
1465	1487	*cis*-Muurola-4(14),5-diene	0.1	0.2	–	–
1475	1491	*trans*-Cadina-1(6),4-diene	1.3	2.1	–	–
1464	1478	9-*epi*-(*E*)-Caryophyllene	–	–	–	0.1
1475	1481	γ-Gurjunene	–	–	–	0.6^∗^
1476	1481	β-Chamigrene	–	–	–	^∗^
1478	1491	γ**-Muurolene**	9.5	13.5	0.4	0.7
1484	1499	**Germacrene D**	–	–	2.5	9.3
1489	1504	β-Selinene	0.5	0.9	0.7	2.2
1492	1496	*cis*-β-Guaiene	–	–	–	tr
1493	1511	*trans*-Muurola-4(14),5-diene	tr	tr	1.0	tr
1496	1512	**Viridiflorene**	5.2	5.8	–	6.6^∗^
1500	1512	Bicyclogermacrene	–	–	–	^∗^
1500	1515	α-Muurolene	–	–	1.9	–
1502	1521	*trans*-β-Guaiene	0.1	0.1	–	–
1505	1519	β-Bisabolene	–	–	0.4	–
1513	1520	γ-Cadinene	0.7	1.1	1.0	1.4
1514	1522	Cubebol	–	–	0.8	–
1520	1525	7-*epi*-α-Selinene	–	–	–	0.1
1522	1529	δ**-Cadinene**	7.1	7.3	–	2.0
1528	1542	**L-Calamenene**	–	–	13.9	–
1528	1542	Zonarene	tr	tr	–	–
1533	1548	*trans*-Cadina-1,4-diene	0.3	0.5	3.1	0.1
1537	1553	α-Cadinene	0.3	0.4	–	0.3
1544	1555	4,5,9,10-Dehydroisolongifolene	–	–	0.7	–
1544	1561	α-Calacorene	0.4	0.5	1.2	tr
1559	1577	Germacrene B	0.9	0.6	–	–
1564	1581	β**-Calacorene**	–	–	0.5	–
1567	1578	Palustrol	–	–	–	3.0
1571	1583	(*Z*)-Dihydro-apofarnesol	0.6	0.3	–	–
1577	1588	Spathulenol	0.1	0.8	–	1.0
1582	1590	**Caryophyllene oxide**	1.2	2.4	18.6	1.3
1592	1604	Viridiflorol	–	–	–	1.5
1600	1609	Guaiol	–	–	–	0.3
1602	1619	**Ledol**	0.1	0.2	–	15.7
1618	1624	1,10-di-*epi*-cubenol	–	–	–	tr
1627	1636	1-*epi*-Cubenol	0.6	0.8	1.2	tr
1638	1650	*epi*-α-Cadinol	tr	tr	–	1.4^∗^
1640	1650	*epi*-α-Muurolol	1.4	1.8	–	^∗^
1644	1654	α-Muurolol	tr	–	1.8	0.2
1645	1663	Cubenol	–	–	2.2	–
1652	1660	α-Eudesmol	–	–	–	tr
1652	1664	α-Cadinol	1.1	1.6	3.8	2.0
1658	1665	Selin-11-en-4α-ol	0.1	tr	–	tr
1658	1670	*neo*-Intermedeol	–	–	–	tr
1665	1675	Intermedeol	–	–	–	0.2
1675	1694	Cadalene	tr	0.2	0.8	–
1676	1699	Mustakone	–	0.1	–	–
1668	1680	14-hydroxy-9-*epi*-(*E*)-Caryophyllene	–	–	–	0.1
1685	1699	Germacra-4(15),5,10(14)-trien-1α-ol	–	0.5	–	0.4
1687	1698	Eudesma-4(15),7-dien-1-β-ol	0.1	–	–	–
1700	1710	Amorpha-4,9-dien-2-ol	0.1	–	–	–
1700	1719	Eudesm-7(11)-en-4-ol	0.1	0.1	–	–
1740	1767	Mint sulfide	0.2	0.1	-	0.1
1843	1847	Hexahydrofarnesyl acetone	0.1	–	–	–
2119	2124	Phytol	1.3	0.2	–	–
Total			95.2	96.8	90.4	97.8

*^a^Those compounds which are in bold represent the main compounds found. ^b^RI lit., retention indices according to Adams (2007). ^c^RI exp., retention indices on RTX-5MS column calculated according to van Den Dool and Kratz (1963). ^d^tr = trace amounts of compound were detected. ^∗^co-eluting compounds.*

**FIGURE 3:**
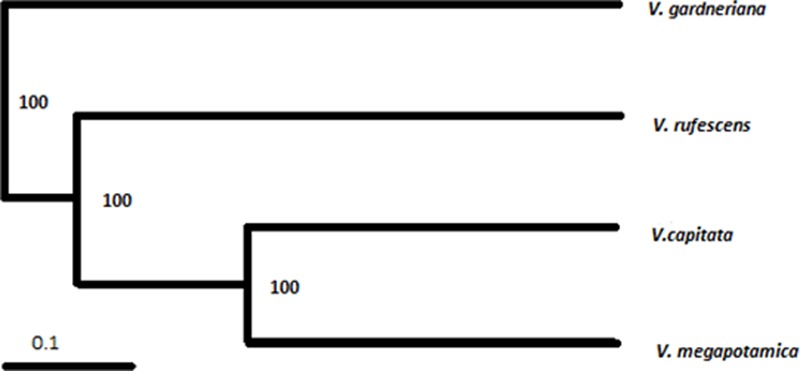
Dendrogram of genetic similarity based on data from Inter-Simple Sequence Repeat profiling using the Jaccard coefficient and the Unweighted Pair Group with Arithmetic Mean method. Numbers indicate values generated with 100,000 bootstrap repetitions.

The 14 ISSR primers generated a total of 90 fragments, 100% of which were polymorphic. The primer with the highest number of fragments was 857 (eleven), while the lowest were 845 and 888 (two). The values of PIC and MI are parameters that allow for estimation of the power of discrimination of the molecular marker by a primer; in our results, the primers 842 and 818 were the most informative (**Table 1**). We observed only fragments to *V. capitata* for primer 823. Genotypes were clustered by UPGMA using the Jaccard coefficient (JC), estimated from the binary data (**Figure 3**). The similarity mean was 0.21 JC (0.08 – 0.45 JC). We observed a clear separation of two groups, with *V. gardneriana* being the most isolated of the four species. In our study, ISSR fingerprints clearly distinguished all of the tested species, with cluster results similar to those performed for the chemical compounds (**Figure 2**).

In the biotoxicity assays, the essential oil extracted from *V. gardneriana* was highly toxic to *A. guerreronis* (LC_50_ 0.8577 mg/mL; CI 0.67-1.15; *x^2^* = 3.17, df = 2, P = 0.20). In contrast, it was not possible to estimate the LC_50_ from *V. capitata, V. megapotamica* and *V. rufescens* essential oils, as increasing concentrations (0.1, 0.5, 1, 1.5, 1.8, 2.0, and 2.3 mg/mL) of these oils did not kill adults of *A. guerreronis* over a period of 24 h. As a way of toxicity comparison, the acaricide abamectin, sprayed at its label rate, inflicted 100% mortality to *A. guerreronis*.

## Discussion

To our knowledge, this is the first report of the chemical composition and biological activity of the essential oil distilled from leaves of *V. capitata, V. gardneriana* and *V. rufescens*. Of the chemical constituents detected, 88.7, 94.5, 80.5, and 90.2% were sesquiterpenes, and 2.3, 0.3, 17.1, and 8.6% were monoterpenes for *V. capitata, V. megapotamica, V. gardneriana* and *V. rufescens*, respectively. Similar chemical compositions were found in the essential oil extracts from *V. capitata* and *V. megapotamica*, where α-copaene, (*E*)-caryophyllene, γ-elemene, α-humulene, gamma-muurolene, viridiflorene and γ-cadinene were the major components (**Figure 2** and **Table 2**). Characterization of *V. gardneriana* revealed that the most abundant compounds were 6,9–guaiadiene (19.3%), caryophyllene oxide (18.6%), L-calamenene (13.9%) and α-cadinol (3.8%). In the case of *V. rufescens*, (*E*)-caryophyllene (21%), ledol (15.7%) and germacrene D (9.3%) were the main constituents detected.

Chemotaxonomy, or chemical variation in a group of compounds, is one of the tools used to define intra- and inter-specific variability in plants (Sena Filho et al., 2007, 2012; Duran-Peña et al., 2015). Our objective was to use the chemical profile in the four *Vitex* species we studied, plus 13 others from the literature, to develop a system for classification of plant specimens derived from this genus. The content of sesquiterpenes with seven and nine members in their ring system in the four *Vitex* species described in this study was remarkable. Recently, genes responsible for sesquiterpene cyclization were evaluated in fungi, which involved scaffolds generated by terpene synthases and cyclases (Quin et al., 2014). Our data indicates that sesquiterpene synthase is likely highly expressed in this genus and hypothesize that this enzyme plays an important role in its chemosystematics. Enzyme mechanisms of sesquiterpene biosynthesis are well known: 5-*epi*-aristolochene synthase (TEAS) found in *Nicotiana tabacum* L. (tobacco) was the first cloned and sequenced sesquiterpene synthase from *Angiospermae* species (Maillea et al., 2006), and could serve as a useful model for understanding how these compounds are built in the plant.

For both the chemical compound and genetic markers evaluated by clustering (**Figures 1, 3**), *V. gardneriana* was isolated from the other three species. This may be related to genotypic differences for this species, as it is found in a different biome (Sena Filho et al., 2008) which may influence the expression of genes used for synthesis of chemical compounds. The use of two data tools (chemical and genetic diversity) more thoroughly supports systematic characterization of the species studied (Rahalia et al., 2016), and validates our chemical marker proposal for the *Vitex* genus, namely, using the sesquiterpene skeleton with seven member rings as a biomarker candidate.

**FIGURE 4:**
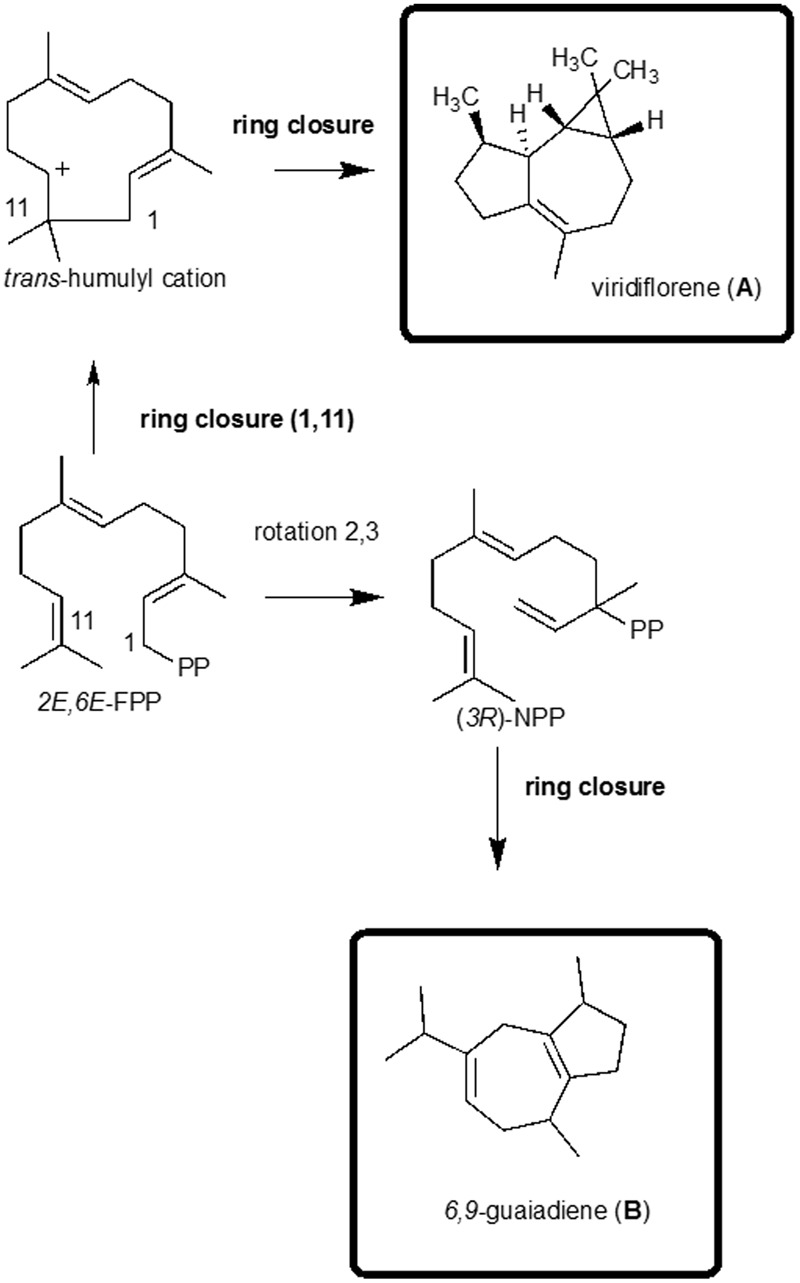
Biosynthesis of the 5,10-cycloaromadendrane type sesquiterpenoids and the seven-member ring compounds found in *Vitex* species viridiflorene **(A)** and 6,9-guaidiene **(B)**.

Interestingly, the chemical structure of the sesquiterpene viridiflorene found in *V. megapotamica, V. capitata* and *V. rufescens* possesses a very unique ring system, similar to 6,9-guaiadiene found in *V. garnderiana* (**Figure 4**). The enzymatic activity of recombinant *Escherichia coli* in TPS 31 from *Solanum lycopenium* led to the discovery of viridiflorine synthase (Bleeker et al., 2011). This compound possesses a similar structure to the africanone scaffold with a 2,6 ring closure (Quin et al., 2014); this type of ring closure could be considered as a more specific signature of the chemoprofile for species in the *Vitex* genus.

In the toxicity bioassays, the essential oil extracted from *V. gardneriana* was highly toxic to *A. guerreronis*, indicating its potential for use in controlling this major coconut pest. On the other hand, it was not possible to estimate the LC_50_ from *V. capitata, V. megapotamica* and *V. rufescens* essential oils. This differential toxicity may be explained by the diversity and amount of sesquiterpenes found only in *V. gardneriana*, such as 6,9-guaiadine (19%), L-calamenene (13.9%), and caryophyllene oxide (18.6%). However, these compounds should be further evaluated concerning the toxicity to *A. guerreronis* using more modern toxicology bioassays. Further, predatory mites, especially those belonging to the family Phytoseiidae, are natural enemies of *A. guerreronis* worldwide (McMurtry and Croft, 1997; Návia et al., 2013). These beneficial mites are naturally found foraging on coconut plants and help to control *A. guerreronis*. Therefore, future research should also assess the acaricidal activity of *V. gardneriana* essential oil to predatory mites aiming at determining its selectivity toward non-target organisms, including pollinators and seed dispersers.

Biological activity of some of the prominent compounds we saw in the *Vitex* species studied here has been defined previously: 6,9-guaiadiene, prominent in *V. gardneriana*, was also found in essential oils extracted from *Bracteosa ruilopezia* (Standl.) Cuatrec and *Baccharis salicifolia* (Ruiz & Pav.) Pers. (Asteraceae)-both oils demonstrated antibacterial activity (Carrizo et al., 2009). It has also been suggested that caryophyllene oxide is toxic; this was evaluated using a direct spray test for females and eggs of the predatory mite *Neoseiulus californicus* (McGregor) (Momen et al., 2014). Further, α-cadinol and caryophyllene oxide were the main volatile compounds found in essential oil extracted from aerial parts of *Teucrium polium* L. – Lamiaceae. The essential oil showed fumigant and repellency effects against the cowpea weevil *Callosobruchus maculatus* F. (Coleoptera: Bruchidae) (Khani and Asghari, 2012; Khani and Heydarian, 2014). α-Cadinol also showed excellent inhibitory action against the termite *Coptotermes formosanus* Shiraki (Isoptera: Rhinotermitidae) (Cheng et al., 2014).

## Conclusion

By combining the volatile profiles and ISSR molecular markers using bioinformatic tools, we were successful in clustering the *Vitex* species in order to identify markers for chemosystematic classification. In this work, *V. gardneriana* was isolated from the other species, expanding our understanding of the chemodiversity of this genus. In addition, the volatile components extracted from *V. gardneriana* showed potential for controlling *A. guerreronis*, as it was highly toxic to this coconut pest.

## Author Contributions

Conceived and designed experiments: JdSF, IB, AT, and HX. Conducted experiments: JdSF and IB. Analyzed data: JdSF, IB, AT, PN, DS, provided reagents: ACdS, AT, and JdSF. Wrote the manuscript: JdSF, AR, ASF, AT, DS, and JD.

## Conflict of Interest Statement

The authors declare that the research was conducted in the absence of any commercial or financial relationships that could be construed as a potential conflict of interest.

## Supplementary Material

TABLE S1 Major components (%) determined in essential oil extracts from 17 *Vitex* species.
